# Monitoring Patient Response to Pembrolizumab With Peripheral Blood Exhaustion Marker Profiles

**DOI:** 10.3389/fmed.2019.00113

**Published:** 2019-05-22

**Authors:** Moira Graves, Giovana CelliMarchett, Belinda van Zyl, Denise Tang, Ricardo E. Vilain, Andre van der Westhuizen, Nikola A. Bowden

**Affiliations:** ^1^School of Medicine and Public Health, Hunter Medical Research Institute, University of Newcastle, Newcastle, NSW, Australia; ^2^Department of Medical Oncology, Calvary Mater Hospital, Newcastle, NSW, Australia; ^3^School of Biomedical Science and Pharmacy, University of Newcastle, Newcastle, NSW, Australia; ^4^Department of Anatomical Pathology, Pathology NSW, Newcastle, NSW, Australia

**Keywords:** melanoma, pembrolizumab, T cell exhaustion, PD-1, TIM-3, LAG-3

## Abstract

Exhausted T cells are effector T cells that are silenced due to continuous T cell receptor (TCR) stimulation from persistent antigens. Characteristics of exhaustion include the increased expression of multiple inhibitory receptors such as programme death-1[PD-1], lymphocyte activation gene 3 [LAG-3], T cell Ig and mucin domain [TIM-3], the loss of effector cytokine secretion and altered transcriptional profile. The PD-1/PD-L1 interaction induces functional exhaustion of tumor-reactive cytotoxic T cells and interferes with anti-tumor T cell immunity. T cell exhaustion has been observed in metastatic melanoma patients where the exhaustion of tumor specific T cells suggests that tumor clearance has been impeded and contributed to tumor immune escape. Checkpoint immunotherapies are antibodies designed to block the interaction between the inhibitory receptors expressed on T cells and their respective ligands. Therapies such as anti-PD-1 (Pembrolizumab and Nivolumab) block these inhibitory receptors and are associated with a significant improvement in overall survival and progression free survival. However, only 20–40% of metastatic melanoma patients experience long-term benefit. In a cohort of 16 metastatic melanoma patients receiving pembrolizumab, blood was serially collected before each infusion (mean 8.3; range 1–12 cycles). The presence of inhibitory markers LAG-3, TIM-3, and PD-1 on the surface of T cells was examined and assessed in relation to patient response to identify if inhibitory markers can be used to differentiate responders from non-responders for Pembrolizumab. We confirmed that across a range of cycles (range 1–26) of pembrolizumab, PD-1 expression was significantly higher on CD4+ T cells from non-responders compared to responders and TIM-3 expressed on the surface of CD8+ T cells was significantly higher in non-responders compared to responders. This longitudinal data confirms previous studies that assessed single timepoints. This study provides preliminary evidence that PD-1 and TIM-3 may be predictive of non-responders when assessed over multiple treatment cycles.

## Background

Antibodies to cytotoxic T-lymphocyte-associated protein 4 (CTLA-4), programme death ligand 1(PD-L1), and programme death 1 (PD-1) have been approved for treatment of metastatic melanoma (1–4).

T cell exhaustion is a process in which there is increased expression of inhibitory molecules, reduced cytokine production, an altered transcriptional programme, metabolic changes and failure to persist long term (5). T cell exhaustion can be viewed as both a mechanism to promote peripheral tolerance and a contributor to cancer. It can dampen the responsiveness of T cells resulting in autoimmunity and establishment host-tumor stalemate. Checkpoint blockade targeting inhibitory receptor pathways (eg PD-1) can reinvigorate exhausted T cells (T_EX_ cells) (6), providing considerable benefits for cancer patients (7, 8). However, recent studies have demonstrated that checkpoint blockade alone does not necessarily improve the durability of “reinvigorated” T_EX_ cells (9). These observations are consistent with the lack of long-term clinical benefits in a proportion of patients upon administration of checkpoint inhibitor blockade (10, 11). Moreover, prolonged chronic infection leads to progressive depletion of T_EX_ cell population (12) and the response to PD-1 blockade also declines over time (13), indicating a poor ability to sustain T_EX_ cell responses. Investigating changes in expression of surface exhaustion markers may indicate in advance if a patient is no longer sustaining a T_EX_ cell response to immune checkpoint inhibitor immunotherapy.

### Exhaustion Markers

T_EX_ cells are induced when there is chronic antigen stimulation such as viral antigens or tumor antigens. The resultant cells express multiple inhibitory receptors including PD-1, CTLA-4, Tim-3, and LAG-3.

PD-1 is a member of the B7-CD28 family and is expressed on myeloid derived cells, B cells and T cells (14). It has two ligands –PD-L1 (expressed on a diverse selection of cells including leucocytes, parenchymal cells and tumor cells) and PD-L2 (expressed by dendritic cells and macrophages). The PD-1 receptor on T cells binds the PD-L1 on the antigen presenting cells (APC) and inhibits the pro-inflammatory scenarios such as T cell proliferation and cytokine production (15). Adaptive resistance to PD-1 monotherapy has been associated with the upregulation of other checkpoint inhibitors (16).

T cell immunoglobulin and mucin domain 3 (TIM-3) is an immunoglobulin (Ig) and mucin domain family cell surface molecule. It was originally found on CD4 helper 1 (Th1) and CD8 T cells and is now known to be expressed on regulatory T (T_reg_) cells, monocytes, macrophages, natural killer (NK) cells, mast cells and dendritic cells (DC). Tim-3 has been designated as an immune checkpoint and exhaustion marker, but recent evidence shows both positive and inhibitory functions (17, 18). Tim-3 has often being co-expressed with other checkpoint inhibitors in T cell exhaustion in tumors in humans (19). In melanoma, the upregulation of Tim-3 along with PD-1 shows a distinctive highly non-responsive population of CD8 T cells (20). Tim-3 is an attractive target for monitoring immunotherapy response as it is an exhaustion marker in both tumors and chronic infections. Studies in mice have shown long term protection when Tim-3 monoclonal antibody was combined with agonist antibodies against the co-stimulatory molecules on CD137 on T cells (21).

Lymphocyte-activation gene 3 (Lag-3) is a cell surface molecule expressed on the surface of activated T cells, NK cells, B cells, and plasmacytoid DC. It has been shown to bind to major histocompatiblity complex (MHC) class II molecules at a site distinct from CD4 and with higher affinity (22). In mice studies, Lag-3 is important for the suppression function of T_regs_ (23, 24). The co-expression of Lag-3 and PD-1 on exhausted T cells or T_regs_ correlates with a greater state of effector T cell exhaustion and suppressive function of T_regs_ (25, 26). Soluble Lag-3 has been detected in the plasma of cancer patients and correlated with higher overall survival and disease free survival rates (27). Clinical trials are now underway to examine the effect of using a Lag-3 mAb (BMS-986016) in cancer patients either as a single agent or in combination with PD-1 blockade (Clinical Trials.gov: NCT02061761, NCT01968109).

The aim of this study was to determine PD-1, Tim-3, and Lag-3 expression on T cell subsets across a prolonged timecourse from patients receiving the anti-PD1 therapy pembrolizumab. Response to the anti-PD1 immunotherapy pembrolizumab was recorded and PD-1, Tim-3 and Lag-3 expression for responders was compared to non-responders.

## Methods

### Cohort

Patients receiving anti-PD1 monotherapy (Pembrolizumab) within the Department of Medical Oncology, Calvary Mater Hospital, Newcastle, Australia were approached to participate in the study. The protocol was approved by the Hunter New England Human Research Ethics Committee (Ethics approval: 14/12/10/4.02). In accordance with the Australian National Statement on Ethical Conduct in Human Research (2007), all subjects gave written informed consent in accordance with the Declaration of Helsinki. A total of 16 patients; 5 females and 11 males, aged from 48 to 80 years gave fully informed, signed consent to participate in the study. The number of cycles of Pembrolizumab received per patient at the start of the serial blood collections ranged from 1 to 26 cycles, with a mean cycle number of 11.4 ± 9.0 (Table 1). Clinical characteristics including age of diagnosis, BRAF mutation status, duration of BRAF/MRK inhibitor treatment, baseline LDH, burden of disease and age at commencement of pembrolizumab was also collated (Table 1). Patients were considered responders if at the time of their last blood collection disease progression defined by iRECIST (28) had not occurred. Non-responders were patients with disease progression confirmed by iRECIST (28), blood collection for non-responders ceased with discontinuation of pembrolizumab. Two study participants had clinically controlled auto-immune diseases (Graves' disease and Sjogren Disease) which was considered in statistical analysis to account for the influence of this on the expression of exhaustion markers.

**Table 1:** Cohort baseline clinical characteristics.

**Patient ID**	**Baseline demographics**											
	**Sex**	**ECOG**	**Age of diagnosis of primary**	**Anatomic location of primary**	**Site of metastatic disease**	**BRAF mutation**	**Duration of BRAF/MEKi**	**LDH baseline**	**M1 status**	**Brain met**	**Burden of disease**	**Age at commencement of Pembrolizumab**
173	F	N/A	70	Vulvar mucosa	LN	No	No	128	a	No	Low	75
205	M	0	75	Leg	Lung	No	No	121	b	No	Low	80
206	F	1	55	Leg	SC, LN	No	No	196	a	No	N/A	61
209	M	0	49	Back	Lung, LN	No	No	160	b	No	Low	55
213	F	0	68	Face	Breast, lung, bone	No	No	167	c	No	N/A	72
216	M	0	72	Toe	Lung, dermal	No	No	153	b	Yes	Low	75
218	M	N/A	No primary	N/A	Adrenal, bone, retroperitoneal LN	Yes	12 m/prog	303	c	Yes	N/A	63
219	M	0	43	Right neck	LN, liver	Yes	9 m/prog	N/A	c	No	N/A	48
220	F	1	No primary	N/A	Lung, pleura, LN, bone, brain, liver	Yes	11 m/prog	313	c	Yes	High	67
254	M	N/A	64	Back	Liver, skin, small bowel	No	No	235	a	No	Low	73
257	F	1	No primary	N/A	Brain, lung	No	No	224	c	Yes	High	63
259	M	N/A	N/A	N/A	Lung, bowel	Yes	15 m/prog	164	c	N/A	N/A	52
266	M	0	40	Sternum	Small bowel, LN	No	No	179	c	No	Low	46
296	M	0	61	Right calf/leg	LN, SC	No	No	N/A	a	No	Low	69
318	M	1	74	Left arm	Brain, adrenal LN, SC	No	No	N/A	c	Yes	High	79
333	M	0	72	Right back	Brain, lung, pleura, rib	No	No	164	c	Yes	Low	75

*N/A, not available; prog, progression; LN, Lymph node; sc, sub-cutaneous*.

Whole blood was collected before each infusion of Pembrolizumab for up to 12 cycles (mean 8.3; range 1–12) or until disease progression. Whole blood was separated into peripheral blood mononuclear cells (PBMCs) and plasma and stored at −80°C.

### Flow Cytometry

PBMC samples were thawed rapidly, washed in RPMI media, rested for 1 h at 37°C and washed twice more in RPMI. The cells were resuspended at a concentration of 1–10 × 10^6^ cells/mL, and 1uL of BD Horizon Fixable Viability stain 575V was added and incubated at room temperature for 15 min. Samples were washed twice with BD Pharmingen Stain Buffer. The following targets were co-stained: Hu CD3 BUV737 UCHT1, Hu CD4 BUV496 SK3, Hu CD8 APC-H7 SK1, Hu CD279(PD-1) BB515 EH12.1, Hu TIM-3(CD366) Alexa 647 7D3, Hu LAG-3(CD223) APC-R700 T47-530 was added to each sample and incubated for 30 min at 2–8^o^C. The samples were washed twice with stain buffer. After final wash, 350 uL of stain buffer was added to each sample and data acquired on a Fortessa X20 (BD Biosciences). Each sample was analyzed in duplicate, non-consecutively.

Gated T-cells (CD3^+^, live, single cell, lymphocytes) were gated as CD4^+^ or CD8^+^ and further divided by presence of PD-1, LAG3, or TIM3. Co-expression of the 3 exhaustion markers was assessed but no further analysis was conducted due to very low percentage of cells positive for 2 or more exhaustion markers. Student's 2-tailed *t*-test was used to compare the mean of each group and one way ANOVA was performed with multiple comparison (Bonferroni) testing between the individual timepoints for each group in our cohort. Statistical analysis was performed on data using FlowJo v12, SPSS V25 and R.

## Results

Sixteen metastatic melanoma patients receiving Pembrolizumab monotherapy participated in this study; 31.3% female and 68.8% male. Five patients ceased treatment due to disease progression (31.3%) during the study. Four patients (25%) were BRAF V600E positive and had received previous BRAF/MEK inhibitor treatment, on which they had progressed before commencing Pembrolizumab (Table 2). Of the 5 patients that progressed on pembrolizumab monotherapy; 2 patients died and 3 patients were enrolled in new clinical trials. There was no significant difference in age at diagnosis (*p* = 0.60) or age when commenced on pembrolizumab (*p* = 0.61) between the responders and non-responders. BRAF mutation status and disease burden (where known) did not correlate with response. There was not a significant difference in PD-1, TIM3, or LAG3 on CD4+ or CD8+ T cells in patients with a BRAF mutation compared to those without.

**Table 2:** Cohort response to pembrolizumab.

**Patient ID**	**Response to treatment**							
	**No of blood samples**	**Cycle no. at start of blood collection**	**Pembrolizumab cycle range**	**Response**	**After cycle**	**Type**	**Progressive disease**	**Responder/ Non-responder**
173	11	3	3–19	PR	4	Metabolic	No	R
205	13	20	20–33	PR/CR	4/22	RECIST	No	R
206	13	23	23–39	PR	4	iRECIST	Yes	NR
209	10	18	18–30	CR	4	RECIST	No	R
213	11	6	6–16	CR	4	Metabolic	No	R
216	12	20	20–31	CR	8	Metabolic	No	R
218	11	6	6–17	PR	4	RECIST	No	R
219	10	26	26–38	PR	4	iRECIST	Yes	NR
220	4	2	2–5	PD	4	iRECIST	Yes	NR
254	11	2	2–13	SD	na	RECIST	No	R
257	1	18	18	PD	na	iRECIST	Yes	NR
259	4	20	20–23	CR	4	RECIST	No	R
266	7	5	5–11	CR	4	Metabolic	No	R
295	7	1	1–8	CR	11	RECIST	No	R
318	5	11	11–15	PR	4	RECIST	No	R
333	2	1	1–2	PD	1	iRECIST	Yes	NR

*CR, complete response; PR, partial response; SD, stable disease; PD, progressive disease; RECIST, Response Evaluation Criteria in Solid Tumors, iRECIST, immune RECIST; Metabolic, response determined by LDH levels: R, responder; NR, non-responder*.

### PD-1

**Figure 1:**
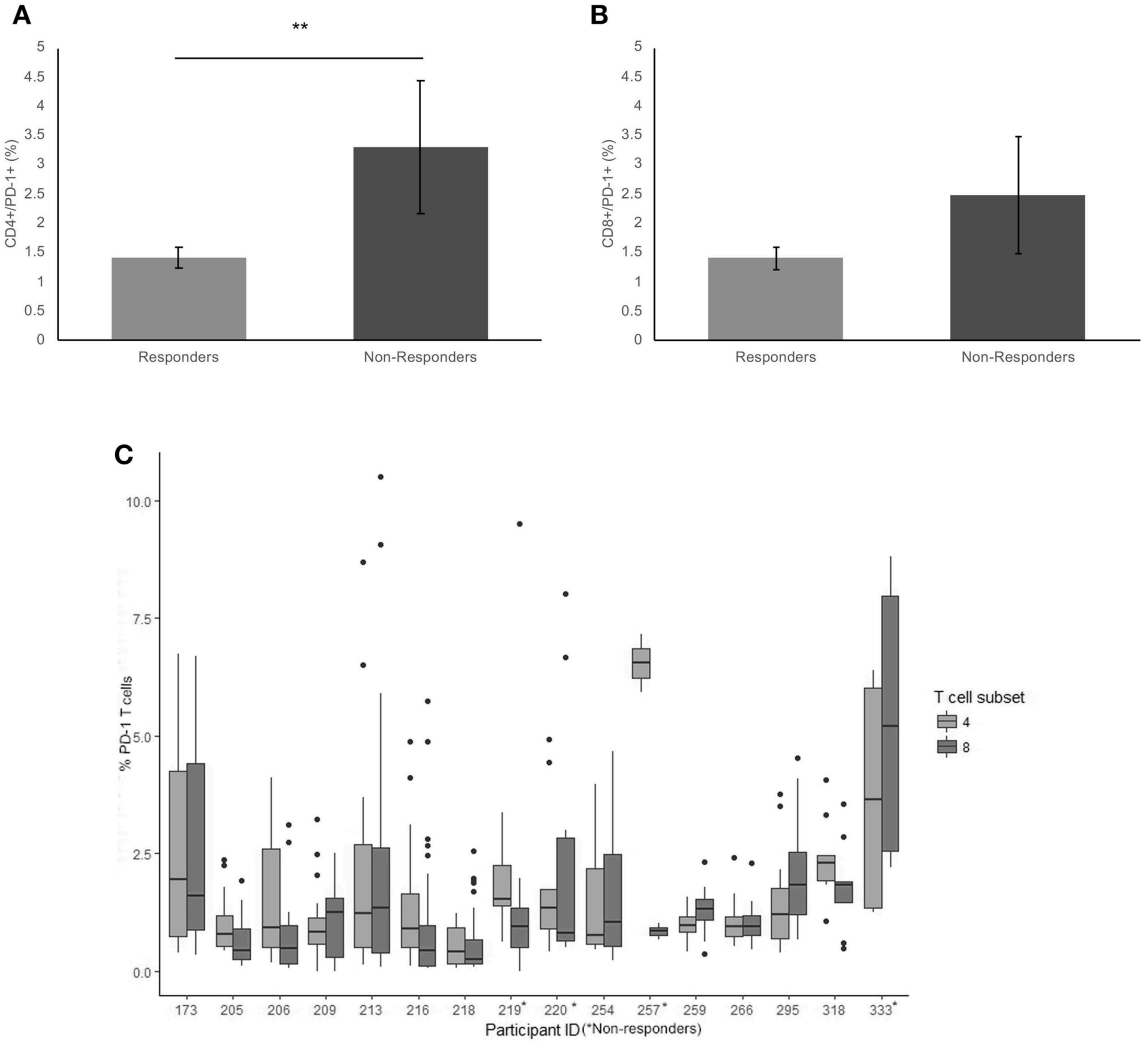
Frequency of PD-1 expressionon CD4+ and CD8+ T cells. **(A)** The mean percentage of CD4+ T cells expressing PD-1 in non-responders responders to pembrolizumab **(B)** The mean percentage of CD8+ T cells expressing PD-1 in non-responders responders to pembrolizumab **(C)** Boxplot of mean frequency of PD-1 on CD4+ and CD8+ T cells per patient per cycle. **p < 0.01.

There was a significantly higher frequency of PD-1 on the surface of CD4+ T cells of the non-responders when compared to the responder cohort (3.3 and 1.4% respectively, *p* = 0.009) (Figure 1A). Examining the average frequency of the PD-1 on the surface of CD8+ T cells in the groups, there was no significant difference between non-responders and responders (*p* = 0.11) (Figure 1B).

PD-1 was detected in all patients on both CD4+ and CD8+ T cells (Figure 1C). The percentage and mean fluorescence intensity (MFI) of CD4+ and CD8+ T cells that were positive for PD-1 show large variability (Figure 1C and Supplementary Figure 1). and one way ANOVA with multiple comparison (Bonferroni) testing between the groups confirmed that there was a significant difference between the frequency of PD-1 on the surface of CD4+ T cells of the non-responders when compared to the responder cohort (*p* = 0.050) when corrected for age of diagnosis, age at commencement of pembrolizumab and number of cycles received. There was no significant difference between responders and non-responders for the frequency of the PD-1 on the surface of CD8+ T cells in individuals. The MFI was not significantly different between responders and non-responders but did show a trend toward the same difference between responders and non-responders.

### TIM-3

**Figure 2:**
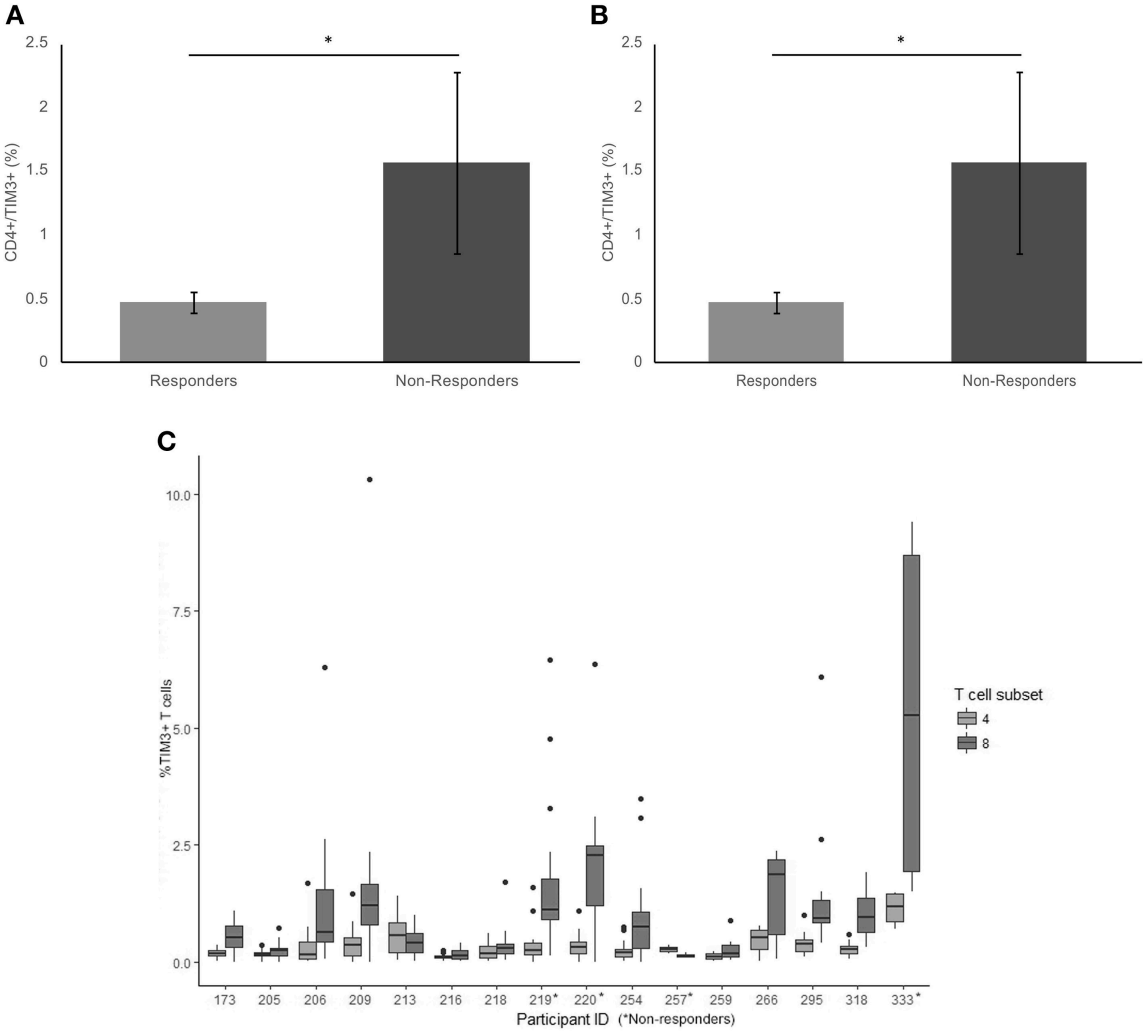
Frequency of TIM3 expression on CD4+ and CD8+ T cells. **(A)** The mean percentage of CD4+ T cells expressing TIM3 in non-responders responders to pembrolizumab **(B)** The mean percentage of CD8+ T cells expressing TIM3 in non-responders responders to pembrolizumab **(C)** Boxplot of mean frequency of TIM3 on CD4+ and CD8+ T cells per patient per cycle. *p < 0.05.

There was a significant difference in average frequency of TIM-3 on the surface of CD4+ T cells between responders and non-responders (*p* = 0.017) (Figure 2A). The average frequency of TIM-3 expressed on the surface of CD8+ T cells was also significantly higher in non-responders compared to responders (*p* = 0.023) (Figure 2B).

TIM-3 was detected at relatively low levels (< 2%, MFI < 80) on CD4+ T cells (Figure 2C and Supplementary Figure 2) in patients at every treatment cycle. Six patients had spiked increase of >5% TIM-3 positive CD8+ T cells, including 3 (75%) of the non-responders (Figure 2B). Statistical comparison between the groups controlling for age of diagnosis, age at commencement of pembrolizumab and number of cycles received confirmed that TIM-3 expressed on the surface of CD8+ T cells was significantly higher in non-responders compared to responders (*p* = 0.047). There was no significant difference between responders and non-responders (*p* = 0.120) in average frequency of TIM-3 on the surface of CD4+ T cells when taking age into consideration.

### LAG-3

**Figure 3:**
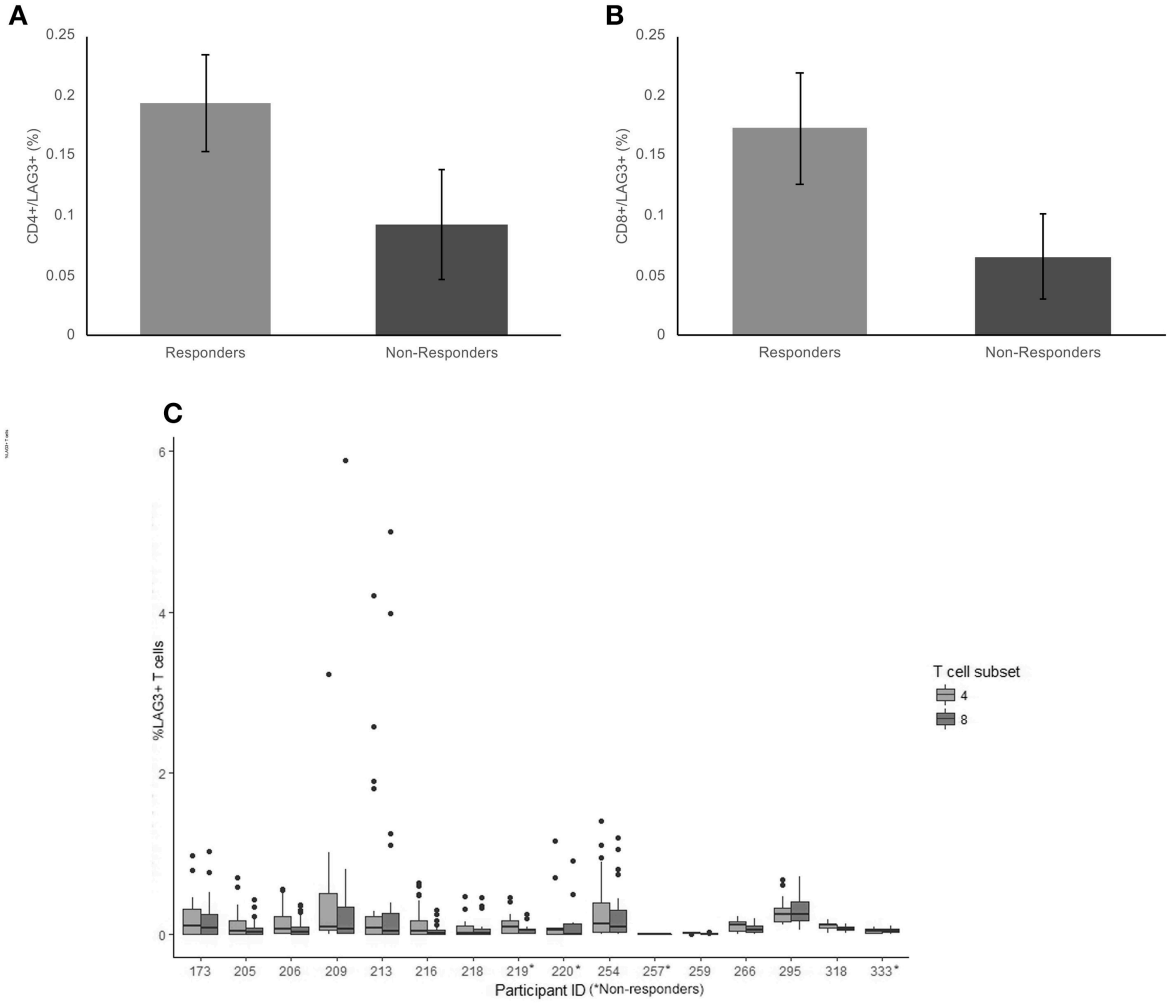
Frequency of LAG3 expression on CD4+ and CD8+ T cells. **(A)** The mean percentage of CD4+ T cells expressing LAG3 in non-responders responders to pembrolizumab **(B)** The mean percentage of CD8+ T cells expressing LAG3 in non-responders responders to pembrolizumab **(C)** Boxplot of mean frequency of LAG3 on CD4+ and CD8+ T cells per patient per cycle.

The average frequency for LAG-3 was very low (< 0.2% average expression across the cohort) There was no significant difference between non-responders and responder groups on CD4+ T cells (*p* = 0.18) (Figure 3A). The average frequency of LAG-3 on CD8+ T cells was also not significantly different between responders and non-responders (*p* = 0.124) (Figure 3B).

LAG-3 was the only marker to not be detected on CD4+ and CD8+ T cells at all cycles. Twelve of the 16 patients had no detectable or < 0.5% positive cells for at least 1 cycle of treatment (Figure 3C). The wide variation in frequency of LAG-3 expression was not related to any other clinical characteristic.

## Discussion

Immune checkpoints are essential for the maintenance of immune self-tolerance. Tumor cells can hijack these immune-regulatory mechanisms allowing cancers to continue to grow and metastasize despite early responses to treatment. The manipulation of these pathways with immune checkpoint inhibitors has given cancers such as melanoma, new and successful treatment options, often with durable responses. Despite this progress, reliable real-time predictive biomarkers of response are still to be identified.

In this study, peripheral blood samples were collected longitudinally to assess the variation in PD-1, TIM-3, and LAG3 during successful treatment and when disease progression occurs. PD-1 was expressed more frequently on the CD4+ T cells of non-responders and TIM-3 was more frequently expressed on both CD4+ and CD8+ T cells of non-responders to Pembrolizumab. LAG-3 showed very low expression and no significant differences between non-responders and responders on either T-cell population.

A recent study by Huang et al. (29) involved a cohort of stage IV melanoma patients receiving pembrolizumab, that were previously treated with Ipilimumab (anti-CTLA-4). The cohort contained 29 patients with 11 responders and 18 non-responders. PBMCs were collected before commencing therapy and every 3 weeks for a total of 12 weeks. PD-1 expression levels were determined by direct staining of PD-1 bound pembrolizumab and TIM-3 and LAG3 expression was determined by commercial antibodies. The percentage of cells positive for PD-1 or TIM-3 expression was similar to the percentage reported here, but there was no significant difference in expression of either marker reported on CD4+ or CD8+ T cells from responders compared to non-responders. The use of the Hu CD279(PD-1) antibody to detect PD-1 in the current study may be responsible for the differing results. The later stages of treatment collected in the current study may also have contributed to the differing results for PD-1 and TIM-3 expression reported.

In another similar study, Krieg et al. (30) examined PBMCs from patients before commencement of pembrolizumab or nivolumab and after 12 weeks of treatment in 2 datasets of (*n* = 10 and *n* = 11). Significantly higher expression of PD-1 was seen on CD4+ T cells from responders in dataset 1 containing 5 responders and 5 non-responders. The difference was not seen in dataset 2. The presence of LAG3 or TIM-3 on the surface of T cells was not investigated. The single timepoint 12 weeks after commencing pembrolizumab, smaller cohort in dataset 1 and much higher percentage of CD4+ cells detected with expression of PD-1 (>10% in dataset 1 compared to < 5% in dataset 2 and the current study) may be the reason for the differing results to those reported herein and dataset 2 in the study by Krieg et al.

The results from direct labeling of pembrolizumb and commercial PD-1 antibody detection methods for quantifying PD-1 have differed between studies (29, 30) Zelba et al. (31) recently compared commercially available anti-PD-1 diagnostic antibodies and tested binding to the PD-1 receptor in the presence of the therapeutic antagonists pembrolizumab and nivolumab. The conclusion of the study was that commercial antibodies to the PD-1 receptor on T-cells do not reliably quantify PD-1 expression levels on PBMCs from patients treated with anti PD-1 antibodies. Interestingly, the only studies to report differences in PD-1 expression on PBMCs have used commercial antibodies to detect PD-1. We hypothesize that an increase in PD1 detection by commercial antibodies such as the BD biosciences Hu CD279(PD-1) MIH4 clone used in the current study may be indicative of an increase in “available” PD1 sites where pembrolizumab has not bound in non-responders. This result is preliminary and requires further investigation and confirmation in a larger cohort, but may be a potential biomarker for non-responders to pembrolizumab.

This is the first study of longitudinally collected blood samples (up to 12 collection timepoints) from patients receiving pembrolizuab. The novel feature of this approach was the analysis of PD-1, TIM3, and LAG-3 before and whilst disease progression was confirmed. It may be difficult to define robust predictive biomarkers for response to Pembrolizumab, given that that immune system is dynamic and there are interpersonal differences in expression between patients. In our study, there was a mixture of long-term responders and patients early in treatment. There was no difference in the age or burden of disease in the responders and non-responders in this study, therefore while these factors contributing to the higher PD-1 and TIM3 expression cannot be completely excluded there was no evidence of age or disease burden influencing the result in this study. Analysis of a validation cohort is required before a peripheral blood biomarker panel of several surface markers, including PD-1 and TIM-3, on a number of immune cell subtypes may be useful from blood samples collected over an extended period of treatment.

## Ethics Statement

This study was carried out in accordance with the recommendations of the National statement on ethical conduct in human research, National Health and Medical Research Council, Australia with written informed consent from all subjects. All subjects gave written informed consent in accordance with the Declaration of Helsinki. The protocol was approved by the Hunter New England Human Research Ethics Committee.

## Author Contributions

MG designed the study, collected, and analyzed data and wrote the manuscript. GC collated clinical information for the cohort. BvZ and DT processed blood samples and contributed to data collection. RV contributed to data analysis, interpretation of clinical information and the final version of the manuscript. AvdW recruited patients, contributed to design of the study, collation of clinical information and the final version of the manuscript. NB designed the study, contributed to data analysis and interpretation, and wrote the manuscript.

### Conflict of Interest Statement

The authors declare that the research was conducted in the absence of any commercial or financial relationships that could be construed as a potential conflict of interest.

## Supplementary Material

Supplementary Figure 1 Mean fluorescence intensity (MFI) of PD-1, expression on **(a)** CD4+ and **(b)** CD8+ T cells in individual non-responders to Pembrolizumab.

Supplementary Figure 2 Mean fluorescence intensity (MFI) of TIM3, expression on **(a)** CD4+ and **(b)** CD8+ T cells in individual non-responders to Pembrolizumab.
