# Supplementary figures and images for: Monitoring Patient Response to Pembrolizumab With Peripheral Blood Exhaustion Marker Profiles
Source: Front Med (Lausanne). 2019 May 22;6:113. doi: 10.3389/fmed.2019.00113 (PMC6540682; doi:10.3389/fmed.2019.00113)

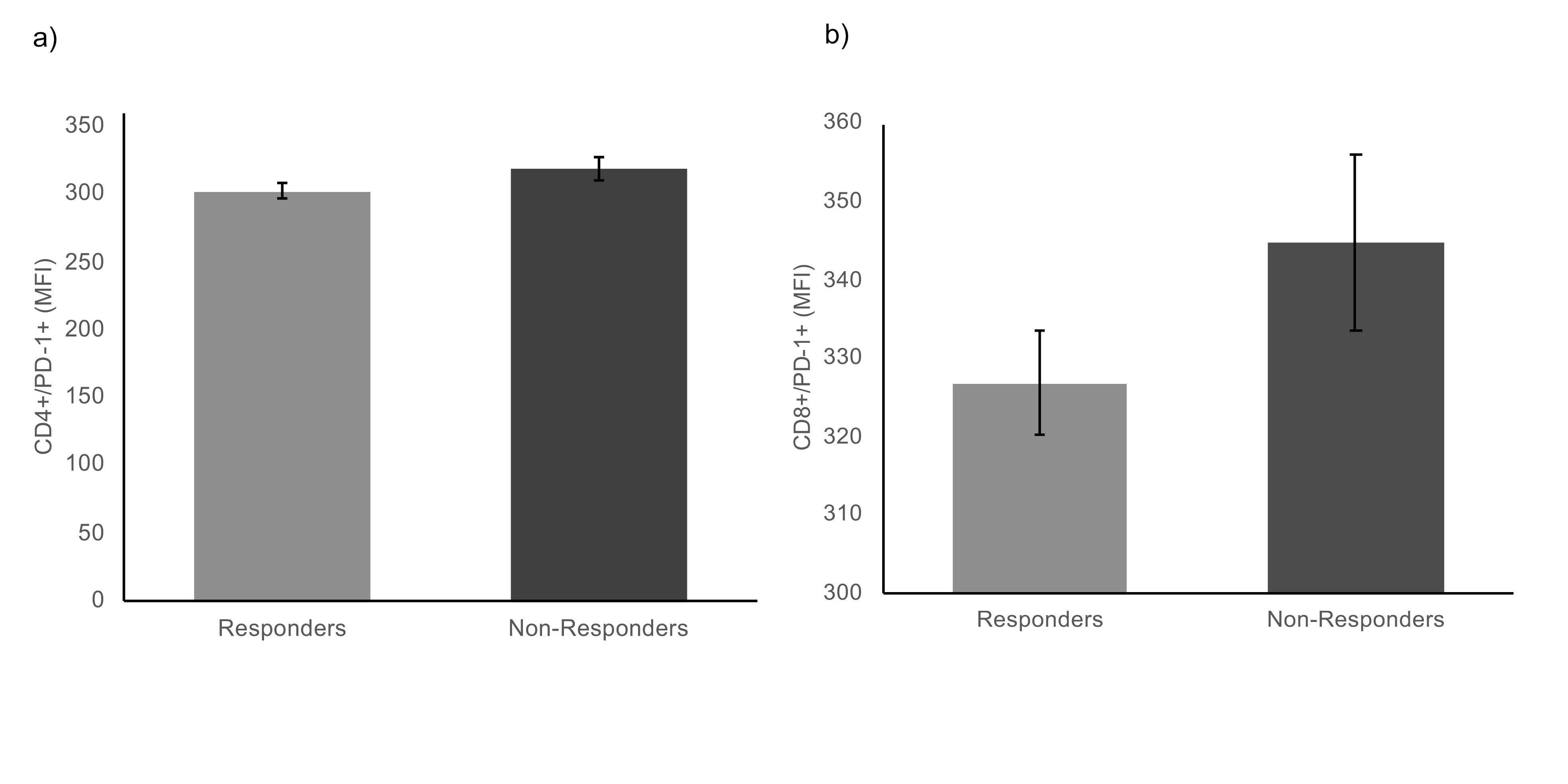

Supplement: Supplementary Figure 1 — Mean fluorescence intensity (MFI) of PD-1, expression on (a) CD4+ and (b) CD8+ T cells in individual non-responders to Pembrolizumab. [file Image_1.JPEG]

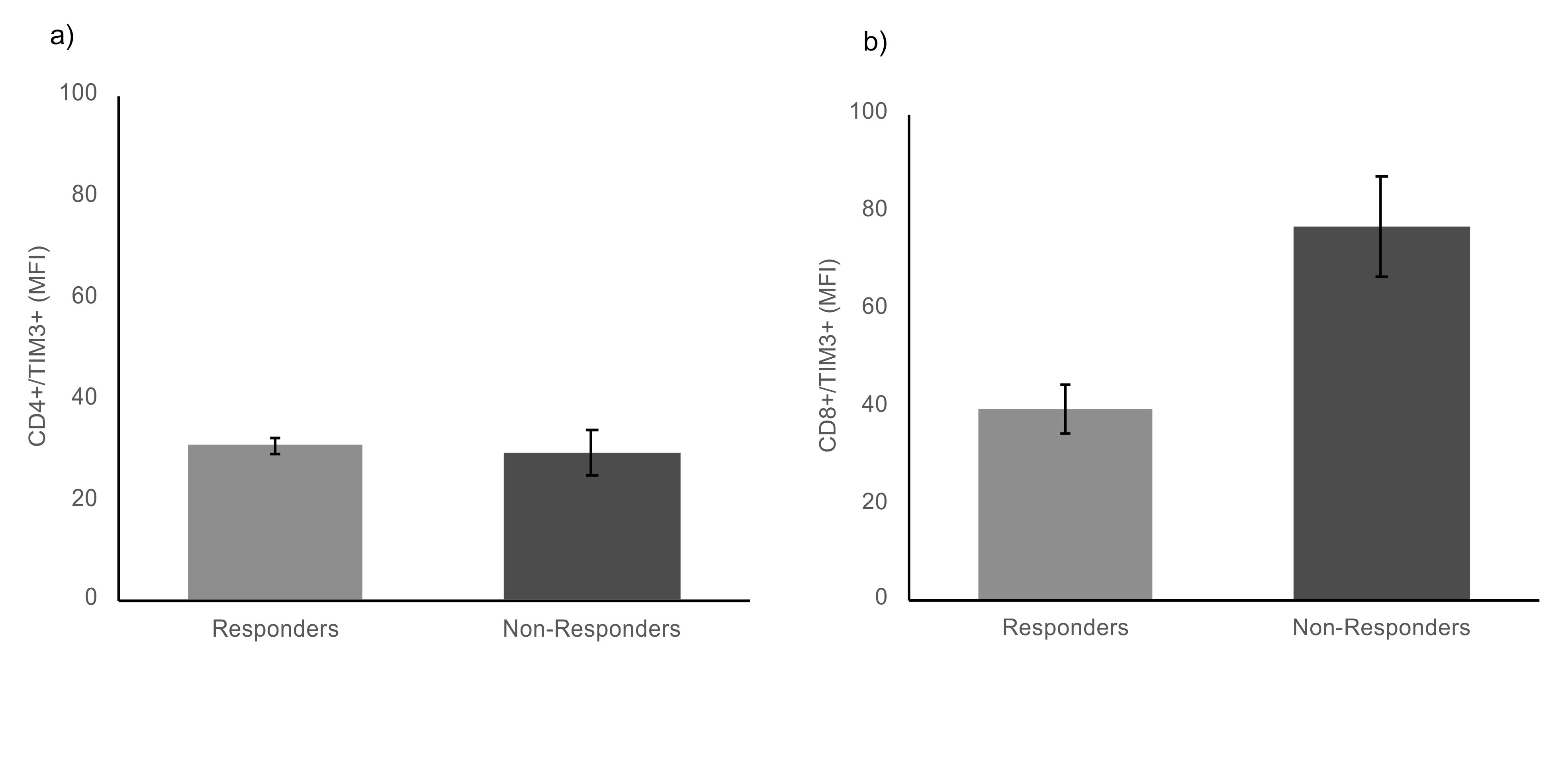

Supplement: Supplementary Figure 2 — Mean fluorescence intensity (MFI) of TIM3, expression on (a) CD4+ and (b) CD8+ T cells in individual non-responders to Pembrolizumab. [file Image_2.JPEG]
